# Comparison of neoadjuvant immunotherapy plus chemotherapy versus chemotherapy alone for patients with locally advanced esophageal squamous cell carcinoma: A propensity score matching

**DOI:** 10.3389/fimmu.2022.970534

**Published:** 2022-10-07

**Authors:** Shao-Wu Jing, Chang Zhai, Wei Zhang, Ming He, Qing-Yi Liu, Ji-Fang Yao, Rui Wang, Zi-Qiang Tian, Jun Wang, Jun-Feng Liu

**Affiliations:** ^1^ Department of Radiation Oncology, Fourth Hospital of Hebei Medical University, Shijiazhuang, China; ^2^ Department of Cardiothoracic Surgery, Fourth Hospital of Hebei Medical University, Shijiazhuang, China

**Keywords:** esophageal squamous cell carcinoma, neoadjuvant chemotherapy, immune checkpoint inhibitors, esophagectomy (or surgery), propensity score matching

## Abstract

**Objectives:**

Clinical studies on immune checkpoint inhibitors (ICIs) combined with neoadjuvant chemotherapy (nCT) have been carried out for the resectable esophageal squamous cell carcinoma (ESCC). So far, few studies have compared the survival outcomes of nCT plus ICIs and nCT alone. This study aimed to compare the efficacy and safety of neoadjuvant ICIs combined with nCT versus nCT followed by esophagectomy for patients with resectable locally advanced ESCC.

**Methods:**

A retrospective analysis of ESCC patients underwent nCT or nCT combined with ICIs followed by esophagectomy (from March 2013 to April 2021) was performed. A 1:1 propensity score matching (PSM) with a caliper 0.01 was conducted to balance potential bias.

**Results:**

A total of 47 comparable pairs of ESCC patients receiving nCT and nCT combined with ICIs were selected for the final analysis. The tumor regression grade (TRG) 0 and pathologic complete response (pCR) rates in the nCT+ICIs group were significantly higher than those of the nCT group (21.7% vs. 4.5%, *P*=0.016; and 17.0% vs. 2.1%, *P*=0.035, respectively). The rate of nerve invasion was 4.3% in the nCT+ICIs group, significantly lower than 23.4% of the nCT group (*P*=0.007). The incidences of adverse events in the nCT+ICIs group were similar compared with the nCT group and there was no grade 5 toxicity in either group. The 1-, 2-year disease-free survival rates (DFS) were 95.7%, 80.7% and 76.1%, 63.8% in the two groups (*P*=0.001, and *P*=0.046, respectively). The 1-year OS was improved in the nCT+ICIs group, which was close to a statistical difference (95.7% vs. 84.8%, *P*=0.074). Local recurrence rate in the nCT+ICIs group was 6.4%, significantly lower than 21.3% of the nCT group (*P*=0.036), while there was no significant difference in the distant metastasis.

**Conclusions:**

Compared with nCT alone, neoadjuvant immunotherapy plus nCT for patients with locally advanced ESCC has an advantage in pathological response, and could improve DFS with a good safety and feasibility, while long term survival validation is still needed further.

## Background

Esophageal carcinoma (EC) ranks seventh in terms of incidence (604, 000 new cases) and sixth in mortality overall (544, 000 deaths) in the world (1). In China, EC is the sixth leading type of cancer and the fourth most common cause of death from cancer, with approximately 320, 000 new cases and 300, 000 deaths in 2020 (2). For resectable locally advanced EC, the CROSS trial and NEOCRTEC 5010 have established neoadjuvant chemoradiotherapy (nCRT) followed by surgery as a cornerstone of the treatment strategy (3, 4). However, a meta analysis involving three randomized controlled trials showed that compared with neoadjuvant chemotherapy (nCT), the overall survival (OS) was not improved in nCRT group (HR: 0.749; 95% CI: 0.397-1.413; P=0.372), although R0 resection and pathologic complete response (pCR) rates are significantly increased (5). A network meta-analysis reveals that nCRT was associated with more postoperative complications and higher postoperative mortality (6). A three-arm randomized phase III study, JCOG 1109, was launched in 2013 to confirm the superiority of preoperative chemotherapy with docetaxel, cisplatin plus 5-fluorouracil (DCF), or chemoradiotherapy with CF (CF-RT) in overall survival over CF for locally advanced esophageal cancer. In 2022, JCOG 1109 demonstrates that nCRT could not significantly improved OS when compared to neoadjuvant CF, whereas DCF did (7). But real-world evidence has been reported that neoadjuvant DCF is not suitable for patients with poor lung function and elderly patients (8). It is urgent to explore novel pattern of neoadjuvant therapy for EC.

O, Reilly et al. (9) reviewed the role of immunotherapy (IO) agents in both early-stage and advanced-stage disease of EC with randomized phase III trials, they proposed that for advanced esophageal squamous cell carcinoma (ESCC) patients, immune checkpoint inhibitors (ICIs) in combination with chemotherapy should be offered in the first-line setting to IO-naive patients regardless of tumor programmed death receptor ligand-1 expression. Recently, a meta analysis revealed that neoadjuvant IO combined with nCT could be used as the recommended therapeutic option (10). However, there is no high quality evidence published for ESCC with neoadjuvant IO, and numerous clinical studies are ongoing for this promising therapy strategy (11, 12). A pilot study including 16 patients diagnosed with locally advanced ESCC had investigated the clinical value and tolerance of neoadjuvant camrelizumab plus paclitaxel and carboplatin, indicated that neoadjuvant IO plus chemotherapy exhibits good efficacy and acceptable tolerance (13). Till now, the most published reports are single-arm clinical trials, few studies compare the survival outcomes of nCT plus ICIs and nCT alone. Huang et al. (14) analyzed efficacy and safety between pembrolizumab combined with chemotherapy and simple chemotherapy in neoadjuvant therapy for ESCC. The pCR and objective response rate (ORR) in the combined group were significantly higher than those of the chemotherapy only group. Nevertheless, we still press for more clinical data including survival outcomes to stand in need of the role of neoadjuvant immunochemotherpay for ESCC. In this study, we aimed to compare the efficacy and safety of neoadjuvant ICIs combined with nCT versus nCT followed by esophagectomy for patients with resectable locally advanced ESCC.

## Patients and methods

### Patients selection

Data of ESCC patients who underwent esophagectomy followed nCT or nCT combined with ICIs in our hospital from March 2013 to April 2021 were retrospectively collected. Inclusion criteria were included: patients with locally advanced resectable stage ESCC; the disease was histopathologically confirmed in tissue samples; receiving simple nCT or plus ICIs following esophagectomy; at least one of the following primary outcomes were reported: R0 resection rate; pCR; incidence of complications and survival. Exclusion criteria included patients with non-resectable tumors or metastases during exploratory surgery; patients receiving other neoadjuvant targeted therapy; patients receiving salvage or palliative surgery. The 8th edition of the International Union Against Cancer/American Joint Committee on Cancer (UICC/AJCC) TNM staging system was used. All procedures were performed in accordance with the 2013 edition of the Declaration of Helsinki. The study protocol was approved by the Ethic Committee of our Hospital. Due to the retrospective nature of the study, informed consent was waived.

### Neoadjuvant treatment regimens

Patients in the nCT+ICIs group received 1-3 cycles of intravenous PD-1 inhibitor (pembrolizumab at a dose of 200mg, camrelizumab at a dose of 200mg, toripalimab at a dose of 240mg, or sintilimab at a dose of 200mg) every 3 weeks and simultaneous chemotherapy consisted of platinum-based drugs and 5-fluorouracil (FP) or docetaxel/paclitaxel (TP). The median usage cycle of neoadjuvant therapy was 2 in this group. Patients in the nCT group received 1-3 chemotherapy (FP or TP regimen) every 3 weeks, and the median usage cycle in this group was 2 either.

### Surgical treatment

For patients suitable for radical esophagectomy after clinical evaluation, the surgery was performed after 4-8 weeks from the end of the last neoadjuvant therapy. Patients received thoracomy esophagectomy, or minimally invasive esophagectomy (MIE), including 2-field or 3-field lymphadenectomy and gastric reconstruction. Two-field lymphadenectomy was regularly conducted, and standard 3-field lymphadenectomy was performed in patients with suspected swollen lymph nodes in the neck.

### Follow-up

The surveillance tests, including physical examination, chest computed tomography (CT), and barium scans were regularly performed during the follow-up, and ultrasonography, endoscopy or positron emission tomography (PET)/CT and magnetic resonance imaging (MRI) were employed if necessary. Toxicity was assessed according to the Common Terminology Criteria for Adverse Events (CTCAE; version 3.0). Surgical complications were recorded within 1 month after resection. All patients were followed up every 3 to 4 months in the first year, followed by an interval of 6 months in the next years.

### Study endpoints

The primary endpoints were the tumor regression grade (TRG), R0 resection rate and pCR rate. The secondary endpoints included toxicities, disease-free survival (DFS), OS, and failure modes. The TRG was evaluated by the proportion of scar and residual tumor, and it was grading into 5 degrees according to Ryan, s TRG system (15): Grade 0 was no residual tumor, grade 1 was residual single tumor cells or small groups of tumor cells, grade 2 was residual partial of tumor, and grade 3 was no regression. pCR was defined as no evidence of residual tumor cells in the primary site and resected lymph nodes of operative specimens. DFS referred to the time from the date of neoadjuvant therapy to the first documentation of recurrence or metastasis. OS was defined as the time from the date of neoadjuvant therapy to death from any cause or lost follow-up.

### Statistical analysis

A one-to-one matching analysis was performed using a caliper width of 0.01 between the nCT+ICIs and nCT groups. The propensity scores were calculated using a logistic regression model, which included age, gender, body mass index (BMI), tumor location, tumor length, clinical stage, surgical procedures and interval from neoadjuvant therapy to surgery. The rates of R0 resection, pCR, complications and failure modes were compared by the Kruskal-Wallis method or the independent-samples t-test. In order to indicate normality of the continuous and categorical variables, the Chi-square test and the Fisher’s exact test were utilized, respectively. The DFS and OS were determined by the Kaplan-Meier method and were compared by the log-rank test. A *P*-value<0.05 was considered statistically significant. The statistical analysis was performed using SPSS 22.0 software (IBM, Armonk, NY, USA).

The propensity score matching (PSM) approach was used for the assembly of a well-balanced cohort using all available explanatory factors (16). Thus, PSM (nCT+ICIs group: nCT group in a 1:1 match) was conducted to adjust the available explanatory factors, including age, gender, BMI, tumor location, tumor length, stage T, stage N, TNM, surgical procedures and interval from neoadjuvant therapy to surgery, which might affect the results.

## Results

### Patients’ baseline characteristics

According to the inclusion and exclusion criteria, 453 patients diagnosed with locally advanced ESCC from March 2013 to April 2021 were enrolled, of whom 48 patients were in the nCT+ICIs group, and 405 patients were in the nCT group. Then, to balance the potential bias, a 1:1 PSM was conducted, and eventually, 47 comparable pairs were matched for the final analysis. The baseline characteristics were listed in **Table 1**. After PSM, the clinical characteristics were well balanced, including age, gender, BMI, tumor location, tumor length, clinical stage T, clinical stage N, clinical stage TNM, chemotherapy regimen, neoadjuvant cycle, surgical approach and interval from neoadjuvant therapy to surgery.

**Table 1:** Baseline characteristics before and after PSM.

Variables	Before PSM			After PSM		
	nCT+ICIs (n)	nCT(n)	*P-*value	nCT+ICIs(n)	nCT(n)	*P*-value
Age (years old)						
≤60	13	181	0.020	13	12	0.815
>60	35	224		34	35	
Gender						
Male	31	287	0.368	30	33	0.510
Female	17	118		17	14	
BMI (kg/m^2^)						
≤22.9	23	208	0.652	22	26	0.409
>22.9	25	197		25	21	
Tumor location						
Upper-thoracic	4	38	0.935	4	1	0.170
Middle-thoracic	37	297		36	43	
Lower-thoracic	7	70		7	3	
Tumor length						
≤5cm	25	236	0.412	25	33	0.090
>5cm	23	169		22	14	
Clinical stage T						
T3	47	400	1.000	47	47	--
T4a	1	5		--	--	
Clinical stage N						
N0	27	225	0.953	26	30	0.661
N1	15	134		15	13	
N2	6	46		6	4	
Clinical stage TNM						
II	26	224	0.176	26	30	0.401
III	21	176		21	17	
IVA	1	5		--	--	
Chemotherapy regimen						
TP	38	333	0.603	37	38	0.797
FP	10	72		10	9	
Neoadjuvant cycle						
1	15	130	0.905	15	23	0.093
≥2	33	275		32	24	
Surgical approach						
Thoracotomy	27	314	0.001	26	33	0.135
MIE	21	91		21	14	
Interval to surgery	44.1±12.9	34.5±15.7	<0.001	40.0±11.2	39.5±10.2	0.766

PSM, propensity score matching; BMI, body mass index; nCT, neoadjuvant chemotherapy; ICIs, immune checkpoint inhibitors; MIE, minimally invasive esophagectomy.

### Neoadjuvant treatment and surgical treatment outcome

Compared with the nCT group, the nCT+ICIs group had an advantage in pathological response. There were 10 cases (21.7%) of TRG 0 in the nCT+ICIs group, 2 (4.5%) in the nCT group, and statistically significant was found (*P*=0.016), seen in **Table 2**. The pCR rate was 17.0% in the nCT+ICIs group and 2.1% in the nCT group (*P*=0.035). The rate of nerve invasion was 4.3% in nCT+ICIs group, which was significantly lower than 23.4% of nCT group (*P*=0.007). The number lymph node removed in the nCT+ICIs group was 23.5 ± 10.9, while 19.2 ± 8.7 in the nCT group (*P*=0.032). Percentage of patients received adjuvant therapy followed surgery was 55.3% (26/47) in the nCT+ICIs group, lower than 76.6% (36/47) of nCT group (*P*=0.03), seen in **Table 3**.

**Table 2:** Comparison of TRG between the two groups.

TRG	nCT+ICIs	nCT	*P*-value
0	21.7% (10/46)	4.5% (2/44)	0.016
1	6.5% (3/46)	4.5% (2/44)	1.000
2	32.6% (15/46)	40.9% (18/44)	0.414
3	39.1% (18/46)	50.0% (22/44)	0.300

TRG, tumor regression grade; nCT, neoadjuvant chemotherapy; ICIs, immune checkpoint inhibitors.

**Table 3:** Surgical treatment outcomes.

Variables	nCT+ICIs	nCT	*P*-value
R0 resection rate	87.2% (41/47)	91.5% (43/47)	0.503
pCR rate	17.0% (8/47)	2.1% (1/47)	0.035
Rate of nerve invasion	4.3% (2/47)	23.4% (11/47)	0.007
Rate of vascular tumor thrombus	6.4% (3/47)	4.3% (2/47)	1.000
Rate of positive lymph nodes	40.4% (19/47)	55.3% (26/47)	0.148
Thoracotom			
Left	80.8(21/26)	78.8% (26/33)	0.851
Right	19.2% (5/26)	21.2% (7/33)	
Lymph node moved number	23.5 ± 10.9	19.2 ± 8.7	0.032
Adjuvant therapy	55.3% (26/47)	76.6% (36/47)	0.030

nCT, neoadjuvant chemotherapy; ICIs, immune checkpoint inhibitors; pCR, pathologic complete response.

### Safety and complications

The complications after neoadjuvant therapy were summarized in **Table 4**. The incidence of bone marrow suppression, rash, myocardial enzyme elevation and transaminase elevation were comparable in both groups (all *P* value>0.05). The majority of patients experienced complications of grade 2 or less, and no grade 5 occurred. The incidence rates of postoperative complications, including anastomotic leakage, pneumonia and gastrointestinal bleeding were also similar in the two groups (all *P* value>0.05), seen in **Table 5**.

**Table 4:** Complications after neoadjuvant therapy.

Variables	nCT+ICIs (n)	nCT (n)	*P*-value
Bone marrow suppression			
Grade 1- 2	13	16	0.503
Grade 3-4	0	0	None
Rash		0	
Grade 1- 2	1	0	1.000
Grade 3-4	0		None
Myocardial enzyme elevation			
Grade 1- 2	5	1	0.206
Grade 3-4	1	0	1.000
Transaminase elevation			
Grade 1- 2	19	13	0.192
Grade 3-4	1	0	1.000

nCT, neoadjuvant chemotherapy; ICIs, immune checkpoint inhibitors.

**Table 5:** Complications after Surgery.

Variables	nCT+ICIs (n)	nCT (n)	*P*-value
Anastomotic leakage	0	3	0.240
Pneumonia	2	1	1.000
Gastrointestinal bleeding	0	1	1.000

nCT, neoadjuvant chemotherapy; ICIs, immune checkpoint inhibitors.

### Follow-up

**Figure 1:**
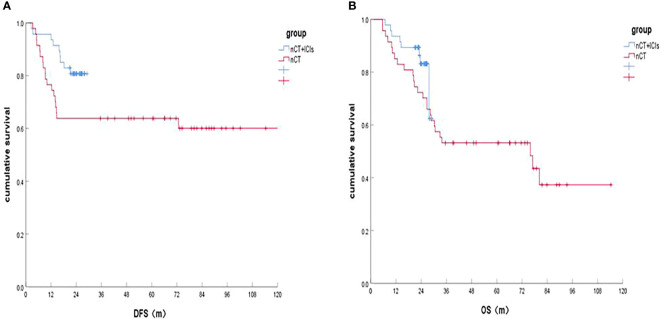
Survival of the nCT+ICIs group and nCT group after follow-up. **(A)** DFS rates between the two groups; **(B)** OS rate between the two groups.

In the two groups, both 1 patient was lost in the follow-Up. The last follow-up time was April 30, 2022. It was shown that the 1-, 2-year DFS rates of the patients in nCT+ICIs group and in nCT groups were 95.7%, 80.7% and 76.1%, 63.8%, respectively (HR, 0.164, *P*=0.001; HR, 0.448, *P*=0.046). In terms of OS, the 1-, 2-year OS rates in the nCT+ICIs group was 95.7%, 83.2% and 84.8%, 72.3% in the nCT group (HR, 0.474, *P*=0.074; HR, 0.564, *P*=0.189). These results were shown in **Figure 1A** and **Figure 1B**.

We analyzed the failure modes of the two groups after surgery, and the results were shown that ESCC patients with recurrence in the nCT+ICIs group was 3 (6.4%), significantly lower than 10 (21.3%) of the nCT group (c^2^, 4.374; *P*=0.036), and the mainly recurrence was regional lymph nodes, while there was no significant difference in the metastasis (c^2^, 0.336; *P*=0.562) between the two groups, seen in **Table 6**.

**Table 6:** Failure modes after radical esophagectomy.

	nCT+ICIs (n)	nCT (n)	χ^2^	*P-*value
Recurrence				
Regional lymph node	2	8	4.374	0.036
Anastomosis	1	2		
Metastasis				
Supraclaviclar lymph node	1	0	0.336	0.562
Abdominal lymph node	2	1		
Liver	2	0		
Bone	0	4		
Lung	1	1		
Brain	0	1		
Subcutaneous	0	1		

## Discussion

JCOG 9907 (17) confirmed the survival benefit of preoperative chemotherapy with CF over post-operative chemotherapy with the same regimen, which had become the current Japanese standard treatment for locally advanced esophageal cancer. Owing to the result, neoadjuvant chemotherapy could be applied as an approach for the treatment of resectable ESCC in China, but the survival benefit of this treatment was still limited. The preclinical studies demonstrated that chemotherapeutic agents could exert immunostimulatory effects, either by activating effector cells and/or inhibiting immunosuppressive cells in the tumor microenvironment or increasing immunogenicity and T-cell infiltration (18–20), a remarkable progress has been recently made in immunotherapy for the treatment of EC. The CheckMate 577 trial (21), of patients with R0 resected esophageal or gastroesophageal junction cancer with residual pathological disease had been conducted to evaluate nivolumab as adjuvant therapy. The median DFS among the patients who received nivolumab was 22.4 months, as compared with 11.0 months among the patients received placebo (HR, 0.69; *P*<0.001).

In the neoadjuvant setting, ICIs is deemed to eliminate micrometastasis and thus lead to superior survival by inducing system immune activation (22). Expansion of tumor resident T cell clones in the peripheral blood had been found in the neoadjuvant immunotherapy (23). In recent years, several studies have reported that nCT combined with ICIs followed by esophagectomy could be recognized as an effective treatment for locally advanced ESCC, and the pCR could be increased to 25%-39.2% (24–27). A multicenter, single-arm, phase II trial aimed to evaluate the safety and efficacy of camrelizumab and chemotherapy as neoadjuvant treatment for locally advanced ESCC had been reported (27). The R0 resection was achieved in 50 (98.0%) patients and pCR was identified in 20 (39.2%). Thirty-four patients (56.7%) had adverse events of grade 3 or worse, with the most common being leukocytopenia, demonstrated nCT combined with IO was a promising neoadjuvant treatment without unexpected safety signals. Up to now, there is no comparative data on the long-term survival between nCT group and nCT combined with ICIs group. In China, a phase III study (HCHTOG1909) comparing neoadjuvant toripalimab plus chemotherapy versus chemotherapy for patients with locally advanced ESCC is in progress (11).

In the current study, 47 pairs of comparable patients with ESCC receiving nCT combined with ICIs and simple nCT were selected for the final analysis after PSM. Compared with the nCT group, the nCT+ICIs group had advantage in TRG and pCR. There were 10 cases (21.7%) of TRG 0 in the nCT+ICIs group, 2 (4.5%) in the nCT group (*P*=0.016). The pCR rates were 17.0%, 2.1% in the two groups (*P*=0.035). In addition, postoperative nerve invasion in the nCT+ICIs group was significantly lower than that of the nCT group (*P*=0.007). The R0 resection rate and the rate of vascular tumor thrombus were similar between the two groups. We observed the adverse effects of the patients. The complications after neoadjuvant therapy and postoperative (including bone marrow suppression, rash, myocardial enzyme elevation, transaminase elevation and anastomotic leakage, pneumonia, gastrointestinal bleeding) were comparable and the incidence of grade 5 was 0 in the two group, indicated that additional neoadjuvant ICIs to nCT was safe and feasible, and it was similar with other studies (4, 28).

To our knowledge, this is the first study to provide 2-year survival on ESCC patients receiving nCT plus ICIs versus nCT alone. The 1-, 2-year DFS rates were both significantly increased in the nCT+ICIs group (*P*=0.001, *P*=0.046, respectively), which might be related to the higher TRG 0 and pCR, lower nerve invasion rate and more lymph node dissections. In terms of OS, there was no significant difference in the 1-, 2-year OS rates between the two groups. However, the 1-year OS improved in the nCT+ICIs group, which was close to a statistical difference (*P*=0.074). Perhaps it was related to the relatively lower pCR rate (17.0%) and small sample size in our retrospective studies. After all, pCR rate was supposed to be strong associated with better survival in ESCC (29, 30). In addition, a more aggressive adjuvant therapy was conducted in the nCT group (*P*=0.03), which probably provided some survival benefits for patients with ESCC.

Regarding the failure modes, our results showed that nCT combined with ICIs could significantly reduce local recurrence (*P*=0.036), but there was no significant difference in terms of metastasis (*P*=0.562), indicated that systemic therapy might be insufficient in the nCT+ICIs group and seemed need to be further strengthened. After all, 83.0% of the patients did not reach pCR in the nCIT group. This also reminds us higher pCR rate is still the focus of the neoadjuvant therapy strategy.

Some imitations were as followed: (1) It was a single-centered retrospective study, due to retrospective nature of the study, the treatment selection bias inevitably existed despite PSM; (2) In view of the almost 10 years span of the included cases, the lymph node dissection in the nCT+ICIs group was significantly more than that of the nCT group [(23.5 ± 10.9) vs. (19.2 ± 8.7), *P*=0.032], which might influence the results potentially; (3) The general information of the enrolled patients lacked data such as PD-L1 expression, so we could not make a detailed assessment of the expression of PD-L1 and the response to neoadjuvant therapy; (4) Lack of large cohort, and the follow-up period was short in the nCT+ICIs group; (5) Regarding times, changes had taken place in surgical techniques and chemotherapy regiments, all of these might affect the final results.

In conclusion, compared with nCT alone, neoadjuvant immunotherapy plus nCT for patients with locally advanced ESCC has an advantage in pathological response, and could improve DFS with a good safety and feasibility, while long term survival validation is still needed further.

## Data availability statement

The raw data supporting the conclusions of this article will be made available by the authors, without undue reservation.

## Ethics statement

The studies involving human participants were reviewed and approved by fourth hospital of Hebei medical university. Written informed consent for participation was not required for this study in accordance with the national legislation and the institutional requirements.

## Author contributions

JW and J-FL conceived the concept and coordinated the design. S-WJ drafted the manuscript with significant contributions from CZ and WZ. MH, Q-YL, J-FY, RW, Z-QT were responsible of visualization, supervision and writing review. All authors listed have made a substantial and direct contribution to the work and approved to publication.

## Funding

This work was supported by Hebei Clinical Research Center for Radiation Oncology (2057702D).

## Conflict of interest

The authors declare that the research was conducted in the absence of any commercial or financial relationships that could be construed as a potential conflict of interest.

## Publisher’s note

All claims expressed in this article are solely those of the authors and do not necessarily represent those of their affiliated organizations, or those of the publisher, the editors and the reviewers. Any product that may be evaluated in this article, or claim that may be made by its manufacturer, is not guaranteed or endorsed by the publisher.
